# Helping the Helpers: Who Assists Old Children of Very Old Parents?

**DOI:** 10.1093/geroni/igaf122.2653

**Published:** 2025-12-31

**Authors:** Sein Kim, Eunju Lee, Kyungmin Kim, Joohyun Kim, Gyounghae Han

**Affiliations:** Seoul National University, Seoul, Republic of Korea; Seoul National University, Seoul, Republic of Korea; Seoul National University, Seoul, Republic of Korea; Seoul National University, Seoul, Republic of Korea; Seoul National University, Seoul, Republic of Korea

## Abstract

With increasing life expectancy, the relationship between very old parents and their “old” children extends. Old children are the main source of support for their very old parents, but they are also likely to experience their own health problems and reduced financial and social resources. Yet, less is known about who helped old children of very old parents in the contexts of prolonged filial caregiving. This study analyzed a sample of 102 older adults (aged 65–72) who have a very old parent (aged 81–97) from the Korean Aging Together Study. This study examined old children’s networks of social support (i.e., size, composition, and assistance type) and how the network characteristics were associated with depressive symptoms and caregiving burden. On average, participants listed two helpers (range = 1–5) as their social support network. The most frequent types of helpers were spouses (67%), siblings (40%), and daughters (27%). The helpers were most likely to provide financial assistance (60%), followed by listening to talk (25%) and emotional support (11%). The majority of participants (70%) included family members only in their networks of social support, while 28% included both family and non-family members. Regression analyses showed that having networks consisting of family and non-family members was related to fewer depressive symptoms and lower caregiving burden, compared to other types of networks. Our findings suggested that it is important to address unique health and social challenges that “older” child caregivers may face while taking care of their very old parents.

